# Urinary Tract Infection and Antimicrobial Resistance Patterns: 5-Year Experience in a Tertiary Pediatric Nephrology Center in the Southwestern Region of Poland

**DOI:** 10.3390/antibiotics12091454

**Published:** 2023-09-19

**Authors:** Anna Kawalec, Justyna Józefiak, Katarzyna Kiliś-Pstrusińska

**Affiliations:** 1 Clinical Department of Pediatric Nephrology, Wroclaw Medical University, Borowska Street 213, 50-556 Wroclaw, Poland; 2 Clinic of Pediatric Nephrology, University Hospital in Wroclaw, Borowska Street 213, 50-556 Wroclaw, Poland

**Keywords:** urinary tract infection, children, antibiotic resistance

## Abstract

(1) Background: Urinary tract infections (UTIs) are among the most common infections in the pediatric population. This study aimed to analyze the urine culture results and antimicrobial patterns over the last 5 years in children diagnosed with UTI. (2) Methods: Retrospective analysis of medical records of 242 patients hospitalized in the Pediatric Nephrology Department diagnosed with a UTI in the years 2018–2022. (3) Results: The most common causative agent was *E. coli,* responsible for 64% of UTIs, followed by *Klebsiella* spp. (16%), *Pseudomonas* spp. (6%), *Enterobacter* spp. (4%), *Proteus* spp. (4%), and *Enterococcus* spp. (3%). Non-*E. coli* UTIs were significantly more frequently observed in patients with congenital anomalies of the kidney and urinary tract or neurogenic bladder and patients receiving antibiotic prophylaxis. For the whole study period, 32% of *E. coli* were resistant to amoxicillin/clavulanic acid, 23.3% to trimethoprim/sulfamethoxazole, 12.2% to ciprofloxacin, and 4.4% to nitrofurantoin. During 2018–2022, the prevalence of *E. coli* resistant to amoxicillin/clavulanic acid varied from 16.7% to 41.2%, and resistance to cefuroxime increased four times (from 4% in 2018 to 16.7% in 2022). Starting in 2021, all isolated *E. coli* strains were classified as susceptible-increased exposure or resistant to cefuroxime. (4) Conclusion: Managing pediatric UTIs remains challenging in clinical practice. The choice of optimal empiric treatment should be considered following local recommendations and individual risk factors assessment and require careful dosage adjustment. Observed changes in antimicrobial resistance indicated the need for frequent updating of local recommendations for the management of pediatric patients with UTIs.

## 1. Introduction

Urinary tract infections (UTIs) are among the most common bacterial infections in the pediatric population [1,2]. Among the risk factors for UTIs are structural kidney and urinary tract anomalies, bowel and bladder dysfunction, neurogenic bladder, immunocompromised states, presence of a foreign body (indwelling catheter or kidney stones), gender, and sexual activity [1,2,3,4]. The most common causative agent for a UTI is *Escherichia coli (E. coli),* responsible for about 70–80% of infections [1,2,4].

Children with congenital anomalies of the kidney and urinary tract (CAKUT) are a particular group of patients who are at high risk of recurrent UTIs, frequent antibiotic therapy, and increasing antimicrobial resistance [1,3,4]. In this group of patients, UTIs are more frequently caused by bacteria other than *E. coli* [1,4].

Infection caused by non-*E. coli* and multi-drug resistant bacteria may be related to previous hospitalizations and patient colonization with pathogens acquired in the hospital environment from other patients, medical staff, or hospital facilities [5,6]. Nosocomial infections most frequently occur in transplant units, neonatal, and intensive care units [7]. The etiology and resistance patterns between community-acquired and hospital-acquired UTIs differ significantly [8]. According to Devrim et al., nosocomial UTIs were most often caused by *Klebsiella pneumoniae* (34.1%) and *E. coli* (26.8%), and more than 70% of isolated bacteria produced the extended-spectrum β-lactamase (ESBL). Also, the resistance rate to meropenem, imipenem, and ertapenem was high [9]. A study by Mongkonsritragoon et al. reported that *E. coli* was a causative agent in 43% of cases of children diagnosed with hospital-acquired UTIs. The resistance rates to third-generation cephalosporin were approximately 75% and to carbapenem nearly 5%. [10]. Hence, apart from the development of infection during hospitalization, stay in a hospital is related to the higher risk of patient colonization with bacteria typically considered nosocomial, such as multi-drug-resistant *Pseudomonas aeruginosa* or *Enterobacteriaceae*-producing ESBL [6,11]. Colonization increases a person’s risk for infection. Thus, the timing of colonization rather than the timing of diagnosis may be essential in determining the origin of resistance in bacteria causing infection [11].

In managing UTIs in the pediatric population, it is essential to promptly start effective antibiotic treatment to improve the patient’s condition, prevent severe complications of UTIs such as bacteremia, and prevent renal scarring [1,4,12]. However, in the scope of increasing resistance to antibiotics observed in bacterial pathogens causing UTI, choosing the best treatment option is a challenge [4,12]. Significant differences exist in the antibiotic resistance patterns of uropathogenic *E. coli* according to the geographical region. Also, individual risk factors for non-*E. coli* infection or infection caused by antibiotic-resistant bacteria should be assessed in each case, such as recent antibiotic therapy, antibiotic prophylaxis, or prior hospitalizations [1]. Therefore, while choosing empirical antibiotic therapy for the treatment of a UTI, recent local recommendations for antibiotic use should be taken into consideration.

This study aimed to analyze the urine culture result and antimicrobial pattern changes over the last 5 years in children diagnosed with a UTI and hospitalized in a tertiary care center for pediatric nephrology in Wroclaw in the southwestern region of Poland. Additional analyses of causative agents and antibiotic resistance patterns in the subgroup of children with CAKUT and children with neurogenic bladder were performed.

## 2. Results

### 2.1. Study Group Characteristic

The final study group consisted of 140 patients diagnosed with a UTI. There were 89 girls (63.57%) and 51 boys (36.43%). Patients’ age varied from 1 month to 17.92 years; the mean age was 5.23 years. For 49 patients (35%), it was the first episode of a UTI. In the whole study group, there were 62 children (44.29%) with CAKUT, 17 (12.14%) with a neurogenic bladder, 30 children (21.43%) underwent urological surgery in the past, and 22 patients (15.71%) received antibiotic prophylaxis because of a recurrent UTI.

### 2.2. Etiology of a UTI

**Figure 1:**
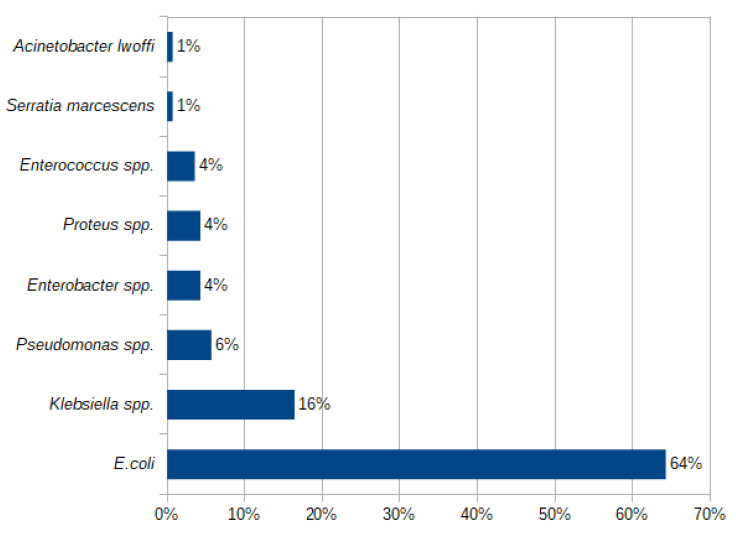
Prevalence (%) of bacterial pathogens causing a UTI in the study group in 2018–2022.

The most common causative agent was *E. coli,* responsible for 64.29% of UTIs in the whole study group. Among non-*E. coli* UTIs, the dominating causative bacteria were *Klebsiella* spp. (16.43%), followed by *Pseudomonas* spp., *Enterobacter* spp., *Proteus* spp., and *Enterococcus* spp. The prevalence of uropathogens identified in all analyzed urine cultures is presented in Figure 1.

We found no significant differences in UTI etiology in children with the first episode of a UTI and those who suffered from UTIs in the past. Non-*E. coli* UTIs were significantly more frequently observed in patients with CAKUT or neurogenic bladder and patients receiving antibiotic prophylaxis (Table 1).

**Figure 2:**
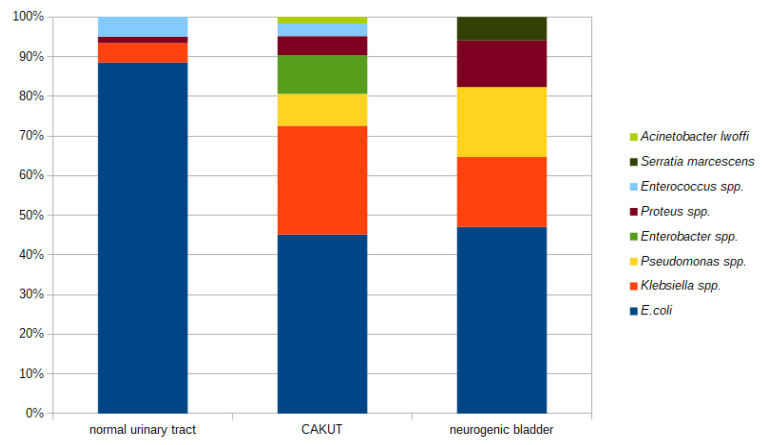
Etiology of a UTI in children with CAKUT or a neurogenic bladder and children without diagnosed abnormalities in the urinary tract.

The prevalence of different etiologic factors for a UTI differed in children with CAKUT or a neurogenic bladder and children without diagnosed abnormalities in the urinary tract (Figure 2).

### 2.3. Antibiotic Resistance Patterns

In the analysis of antibiotic resistance patterns, we focused on *E. coli* and *Klebsiella spp.*, as these two were the most common uropathogens in all analyzed urine cultures. The susceptibility categories, according to the definitions proposed by the European Committee for the Study of Antimicrobial Susceptibility (EUCAST) in 2019, were used and were as follows: “susceptible”, “susceptible-increased exposure”, and “resistant”. The category “susceptible-increased exposure” replaced the previously used term “intermediate” and indicates that to achieve clinical success, the antimicrobial dosage should be adjusted, and a higher concentration of a drug at the site of infection is required [13].

#### 2.3.1. Antibiotic Resistance Patterns for *E. coli*

For the whole study period, 32.22% of *E. coli* strains were resistant to amoxicillin/clavulanic acid, 23.33% to trimethoprim/sulfamethoxazole, 12.22% to ciprofloxacin, and 4.44% to nitrofurantoin. Resistance to cefuroxime was found in 7.77% of cultured *E. coli,* and 34.44% of strains were classified as susceptible-increased exposure. The resistance rate to third-generation cephalosporins (cefotaxime, ceftazidime, and cefixime) was 10%. Six strains producing extended-spectrum β-lactamases (ESBL) were found in all *E. coli*-positive urine cultures (6.66%).

**Figure 3:**
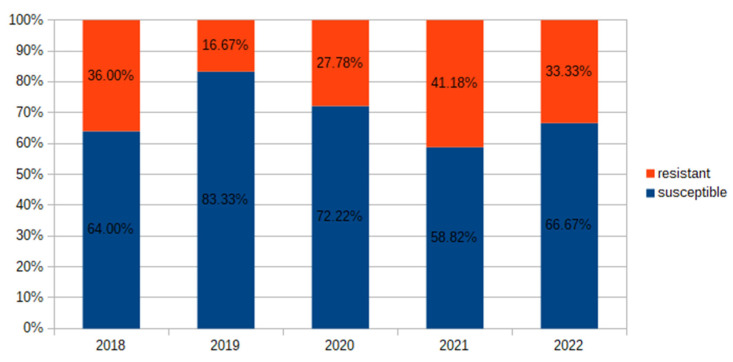
Prevalence (%) of *E. coli* resistant to amoxicillin/clavulanic acid in 2018–2022.

**Figure 4:**
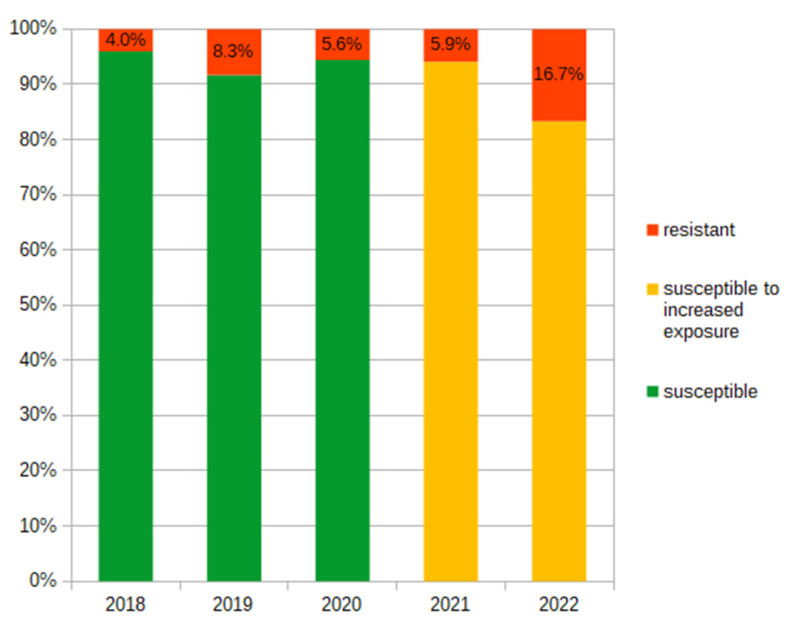
Changes in prevalence (%) of *E. coli* resistant to cefuroxime and susceptible-increased exposure to cefuroxime in 2018–2022.

During 2018–2022, we observed that the prevalence of *E. coli* resistant to amoxicillin/clavulanic acid varied from 16.67% to 41.18% (Figure 3). The prevalence of resistance of *E. coli* to cefuroxime increased four times in the analyzed period (from 4% to 16.67%). Also, starting in 2021, all isolated *E. coli* strains were classified as either susceptible-increased exposure or resistant to cefuroxime (Figure 4).

#### 2.3.2. *E. coli* Antibiotic Resistance in Patients with No Abnormalities in the Urinary Tract, with CAKUT and a Neurogenic Bladder

**Figure 5:**
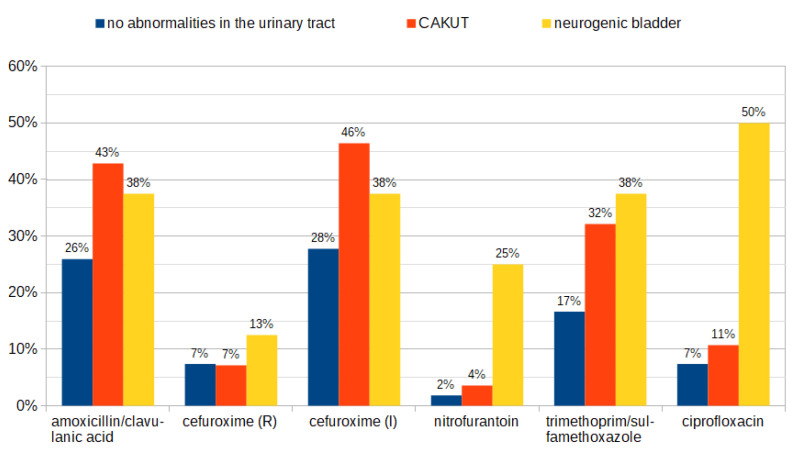
Resistance patterns of *E. coli* isolated from patients without abnormalities in the urinary tract, in children with CAKUT, and with a neurogenic bladder. R—resistant; I—susceptible-increased exposure.

The prevalence of *E. coli* strains resistant to amoxicillin/clavulanic acid was the highest among patients with CAKUT (43%) in comparison to children with a neurogenic bladder (38%) and patients with no abnormalities in the urinary tract (26%). Similar discrepancies between groups were observed for *E. coli* isolates classified as susceptible-increased exposure to cefuroxime. For trimethoprim/sulfamethoxazole, ciprofloxacin, and nitrofurantoin, the highest percentage of resistant *E. coli* was identified in groups of children with a neurogenic bladder (38%, 50%, and 25%, respectively) and those with CAKUT (32%, 11%, and 4%, respectively). Resistance patterns of *E. coli* isolated from different groups of patients are presented in Figure 5.

#### 2.3.3. Antibiotic Resistance Patterns for *Klebsiella* spp.

The *Klebsiella* spp. accounted for 16% of UTIs in the study group. The prevalence of strains resistant to amoxicillin/clavulanic acid was 43.48% and to ampicillin 21.74%. For cephalosporins, the highest resistance rate was for cefuroxime (30.43%) and cefotaxime (30.43%), followed by cefepime (21.74%), ceftazidime (13.04%), and cefixime (13.04%). There were 26.09% of *Klebsiella* spp. isolates resistant to trimethoprim/sulfamethoxazole. Among all *Klebsiella* spp. isolates, six strains (26.1%) producing extended-spectrum β-lactamases (ESBL) and one strain (4.4%) producing AmpC β-lactamases (AmpC) were identified.

### 2.4. Antibiotic Resistance Risk Factors Analysis

To assess the risk factors of a UTI caused by antibiotic-resistant bacteria, a multiple regression model was applied. Analyzed risk factors were age, gender, first episode of a UTI vs. more than one UTI in the past, diagnosed CAKUT or a neurogenic bladder, previous antibiotic therapy for a UTI or other infections, antibiotic prophylaxis against UTIs, and past urological surgery.

The results show a significant regression equation (F = 2.629, *p* < 0.008, ∆R2 = 0.954). The model revealed that a neurogenic bladder and urological surgery in the past are positively correlated with UTIs caused by antibiotic-resistant bacteria (*p* < 0.05). In contrast, there was a negative correlation between previous antibiotic prophylaxis and a UTI caused by resistant uropathogens (*p* < 0.05). The results of multivariate regression are presented in Table 2.

### 2.5. Prescribed Antibiotic Therapy

**Figure 6:**
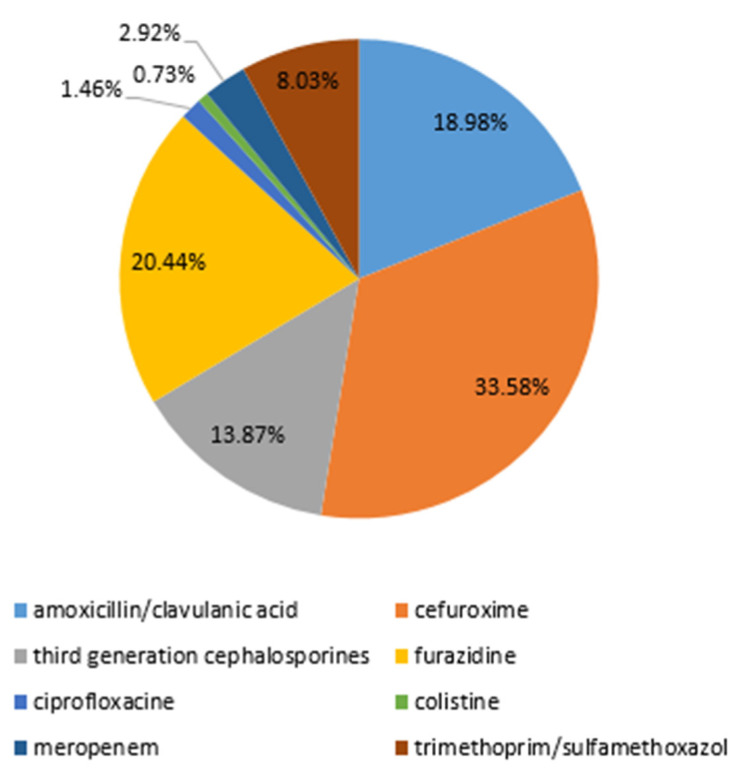
Administered antibiotic therapy chosen empirically or continued if started in other hospitals.

In general, the most frequently used antibiotic chosen empirically or continued if started in other hospitals was cefuroxime (33.58%), followed by furazidine (20.44%), amoxicillin/clavulanic acid (18.98%), third-generation cephalosporines (13.87%), and trimethoprim/sulfamethoxazole (8.03%) (Figure 6).

In 36 cases (25.71%), the treatment needed to be changed and adjusted according to the antibiogram. The most common uropathogen isolated in these cases was *E. coli,* identified in 58.33% (21) urine cultures. Among this group of patients, there were 9 (25%) children with a first episode of a UTI, 15 (41.17%) children with CAKUT, and 7 (19.44%) children with a neurogenic bladder. A history of previous antibiotic therapy for a UTI or other infections was positive in 22 patients (61%), and 7 patients (19.44%) received antibiotic prophylaxis because of a recurrent UTI.

## 3. Discussion

UTIs in children are a common problem in clinical practice. Involvement of renal parenchyma may lead to inflammation and renal scarring, with long-term consequences such as hypertension and impaired renal function [1,3,14]. Thus, proper management of a UTI includes promptly starting empiric antibiotic therapy, which is essential to treat acute infection and prevent its possible complications [1,3,4,14].

The most common causative agents for a UTI in our study were *E. coli* and other enteric bacteria, such as *Klebsiella* spp., *Proteus* spp., *Enterococcus* spp., and *Enterobacter* spp., which is in line with previous observations [1,4,12,14]. Our study confirmed that children with CAKUT and a neurogenic bladder are particular groups of patients prone to UTIs caused by non-*E. coli* bacteria and uropathogens characterized by higher rates of resistance to different antibiotics [1,3,4,12,14,15,16,17]. Nevertheless, the most common causative agent for a UTI in children with CAKUT is *E. coli* [15,18].

We found no significant differences in UTI etiology in children with a first episode of a UTI and those who suffered from UTIs in the past. A relatively high percentage of non-*E. coli* infections in children with the first episode of a UTI may be related to the characteristics of this subgroup, in which 1/3 of patients were children with CAKUT, and 8.16% underwent urological surgery in the past. When choosing the optimal therapy for UTI, local recommendations for antibiotic therapy and individual assessment of each patient’s risk factors for non-*E. coli* and drug-resistant bacteria infection should be carefully evaluated [1,3,14,19].

In different European regions, the percentage of *E. coli* strains isolated from pediatric patients with a UTI resistant to amoxicillin/clavulanic acid varied from 12.2% in Greece [20] to approximately 25% in Italy [21] and Spain [22]. Hrbacek et al. (2020) reported resistance of *E. coli* to amoxicillin/clavulanic acid of 10% in Central Europe in urine cultures of adult patients hospitalized in the urology department [23]. The alarming trend of increasing antibiotic resistance of *E. coli* in pediatric patients diagnosed with a UTI within a relatively short period and with a velocity of approximately 2% per year was reported by Dejonckeheere et al. [24].

In our study, the prevalence of *E. coli* resistant to amoxicillin/clavulanic acid was 32%. The rising problem of the resistance of *E. coli* to amoxicillin/clavulanic acid is highlighted in the updated Recommendations of the Polish Society for Pediatric Nephrology regarding the management of children with urinary tract infections published in 2021 [19]. According to the recommendations, amoxicillin/clavulanic acid in treating a UTI should be considered only for confirmed susceptibility [19]. The study conducted in the northeastern region of Poland among children diagnosed with a UTI reported the prevalence of *E. coli* resistant to amoxicillin/clavulanic acid at 24.8% [25]. On the other hand, the resistance to amoxicillin/clavulanic acid in the southern region of Poland in the adult population reached 49.9% [26]. These data indicate that the susceptibility to antibiotics may vary significantly between regions within one country. In the study group, it is noteworthy that the number of children hospitalized in the past and probably colonized with hospital-acquired pathogens is relatively large. This might partially explain the origin of a high rate of *E. coli* isolates resistant to amoxicillin/clavulanic acid.

We identified 7.7% of *E. coli* isolates resistant to cefuroxime. According to recent European studies in pediatric patients with a UTI, the rate of *E. coli* resistant to cefuroxime ranges from 14.5% in Germany [27] to 19% in Ukraine [28]. In Greece, the resistance rates for first and second-generation cephalosporines were 3.3% for cefoxitin and 28.8% for cephalothin [20]. Regarding the resistance of *E. coli* to third-generation cephalosporines, our results align with data from other Polish regions [25,29].

Several recently published studies describe the alarming trend of decreasing sensitivity to antibiotics [22,24,25,28,30,31,32]. We observed a significant growth in the percentage of *E. coli* strains classified as susceptible-increased exposure or resistant to cefuroxime. Similarly, Budnik et al. reported that sensitivity to cefuroxime was confirmed in only every second child with a UTI [28]. Although the sensitivity to third- and four-generation cephalosporins remains relatively high [21,28,29,32], some studies revealed increasing resistance with an estimated rate of 1% per year [24]. This trend may have a significant implication for clinical decisions in the future. Recent Polish recommendations in the management of acute pyelonephritis in children older than 3 months indicate second- or third-generation cephalosporines or amoxicillin/clavulanic acid (only in case of confirmed sensitivity) or ciprofloxacin (as “emergency” therapy in a clinically justified situation) as the first-choice antibiotic [19]. Changes in antimicrobial resistance patterns in a relatively short period suggest the need to frequently update local recommendations for empiric antibiotic therapy in UTIs.

In ¼ of cases, there was a need to change the antibiotic therapy chosen empirically or continue if started in other hospitals. According to the literature, the rate of inadequate empirical antibiotic therapy in pediatric patients with a UTI in different centers varied from 5 to 22% [33,34]. The risk of prescribing empirical antibiotics to which the cultured pathogen was later found resistant at an antibiogram was higher in cases of a UTI caused by *Klebsiella* spp., *Enterobacter* spp., and mixed organisms [34]. The clinical outcome of inadequate antibiotic therapy differed between antibiotic groups [33]. Among the most frequently chosen initial antibiotic therapy, a combination of penicillin and β-lactamase inhibitors presented a high failure rate and was ineffective in 57% of cases [33]. Despite the proven resistance of bacteria in urine culture, clinical improvement was observed in more than half of pediatric patients with UTIs on discordant therapy [33]. Jerardi et al. showed no significant differences in time of fever resolution between patients with UTI on inadequate and adequate antibiotic therapy [34]. Wang et al. assessed the clinical response in discordant therapy in children treated for UTIs caused by pathogens resistant to third-generation cephalosporins and reported clinical improvement in more than 80% of cases [35]. These findings indicate that despite the resistance of an isolated uropathogen, empiric therapy might still be clinically effective. Nevertheless, before starting the initial antibiotic therapy for a UTI, clinicians should search for and carefully evaluate all the factors that may determine the optimal choice of first-line antibiotic treatment.

The results of the multiple regression model were performed to identify risk factors for a UTI caused by resistant bacteria and showed that a neurogenic bladder and urological surgery in the past are positively correlated with a UTI caused by antibiotic-resistant bacteria. Our results also showed that UTIs in children with CAKUT and a neurogenic bladder are more frequently caused by non-*E. coli* bacteria. Based on our findings, we would suggest avoiding amoxicillin/clavulanic acid as an empirical treatment of patients with CAKUT and a neurogenic bladder due to the resistance rate of *E. coli* of approximately 40% and ciprofloxacin in patients with a neurogenic bladder (isolated *E. coli* strains resistant in 50%). In both groups, the dose of cefuroxime should be carefully adjusted, as the percentage of *E. coli* classified as susceptible to increased exposure reached 38–46%. These findings might be a suggestion when choosing the empirical therapy for a UTI in patients with CAKUT or a neurogenic bladder. Past antibiotic therapy and results of previously performed urine cultures might also be helpful in the decision-making and should be considered.

Another problem in the management of UTIs is the prevention of recurrent infections in particular groups of patients. There is no consensus regarding the prescription of antibiotic prophylaxis. According to the literature, the incidence of a UTI caused by resistant bacteria in children receiving antibiotic prophylaxis has doubled in the last few years [15]. Recent studies search for effective non-antibiotic prophylaxis methods, including dietary supplements, probiotics, immuno-stimulants, and vaccines [36,37,38,39]. However, more research is required to draw evidence-based recommendations for preventing recurrent UTIs in clinical practice.

This study presents several limitations. Its retrospective character is connected with a risk of missing or incorrect data. The study group is relatively small. As data were collected in a tertiary center for pediatric nephrology, the percentage of children with diagnosed kidney and urinary tract abnormalities in the study group is relatively higher than in the general population. In a tertiary center, the percentage of children who were hospitalized before, including hospitalizations due to previous UTIs, urological procedures, or to conduct a diagnostic of the urinary tract, is higher than in a regional hospital, and patient colonization with hospital-acquired bacteria may result in differences in the etiology of a UTI and bacteria resistance. Nonetheless, the antimicrobial patterns we observed reflect the antibiotic resistance of uropathogens in our region, and additional analysis of urine culture isolated from particular groups of patients might be useful in everyday clinical practice.

## 4. Materials and Methods

A retrospective analysis of medical records of patients aged from 1 month to 18 years old hospitalized in the Pediatric Nephrology Department at the Wroclaw University Hospital diagnosed with UTI in 2018–2022 was performed. Inclusion criteria were a diagnosis of a UTI according to the ICD-10 code N39.0 or N39.8 (in the case of N39.8, descriptive diagnosis of a UTI was required) confirmed by the presence of leukocyturia and significant bacteriuria in urine culture. We collected demographic data such as age and gender, present and past medical history, and laboratory test results, including urine culture. For each positive urine culture, we collected the following data: microorganism identified, number of colony-forming units per mL (CFU/mL), mechanism of resistance if present, and antibiotic sensitivity profile. The urine sample collection methods used for the urine culture in the Clinical Department of Pediatric Nephrology at Wroclaw Medical University are clean-catch, midstream, or catheterization.

After the initial data search, we identified 242 records of children with ICD-10 codes of N39.0 or N39.8 and descriptive diagnoses of a UTI. We excluded from the analysis 67 cases of patients with negative urine culture or no urine culture available (patients who were referred to our center to continue treatment and diagnostics with antibiotic therapy for a UTI started prior to admission to our center), 3 patients with fungal urinary tract infection, 15 patients with a mixed urine culture, 16 patients who were hospitalized to conduct the diagnostic process due to recurrent UTIs, and 1 asymptomatic patient with a neurogenic bladder with a positive urine culture before urological procedure. For the final analysis, 140 cases were included (Table 3).

In the final study group, 111 patients (79.29%) were referred to the hospital because of symptoms suggesting a UTI, 9 patients (6.43%) because of the incorrect result of control urine tests performed in ambulatory settings, and 20 children (14.29%) to conduct or continue diagnostic toward CAKUT or other disorders of the urinary tract. In the latter group of patients, the diagnosis of a UTI was based on the results of urine samples collected during the admission to the hospital ward (in the case of leukocyturia in the urinalysis, a urine sample was collected and cultured). None of the diagnosed UTIs were classified as nosocomial infections, defined as infections acquired during the process of care (including prevention, diagnosis, or treatment) and not present or incubating at admission.

Collected data were inserted into a database with anonymity secured. Statistical analysis was performed with the use of Microsoft Excel and STATISTICA software. A comparison of UTI etiology between particular subgroups of patients was conducted using the chi-square test. The multiple regression model was used to assess the possible risk factors related to UTIs caused by resistant bacteria. The significance level was assumed as *p* < 0.05.

## 5. Conclusions

Although UTIs are among the most common bacterial infections in children, their management in clinical practice remains challenging. Children with CAKUT and a neurogenic bladder are particular groups of patients with a higher prevalence of a UTI caused by non-*E. coli* bacteria and antimicrobial-resistant pathogens. Nevertheless, *E. coli* remains the most common causative agent of UTIs in children and those diagnosed with CAKUT. The choice of optimal empiric treatment should be considered in accordance with local recommendations and individual risk factors assessment in each patient. We observed an alarming trend of an increasing percentage of *E. coli* strains classified as resistant or susceptible-increased exposure to second-generation cephalosporins, among the most commonly chosen first-line therapies for febrile UTIs. This implies careful dosage adjustment and suggests the need for frequent updates of local recommendations for the management of pediatric patients with UTIs.

## Figures and Tables

**Table 1:** Etiology of a UTI in different subgroups of patients.

Patients’ Subgroup	Number of Patients in the Subgroup	*E. coli*	non-*E. coli*	*p* Value
First episode of UTI	49	36 (73.5%)	13 (26.5%)	0.096
>1 UTI in the past	91	54 (59.3%)	37 (40.7%)	
CAKUT	62	28 (45.2%)	34 (54.8%)	<0.001
No abnormalities in urinary tract	61	54 (88.5%)	7 (11.5%)	
Neurogenic bladder	17	8 (47.1%)	9 (52.9%)	<0.001
No abnormalities in urinary tract	61	54 (88.5%)	7 (11.5%)	
Antibiotic prophylaxis	22	6 (27.3%)	16 (72.7%)	<0.001
No antibiotic prophylaxis	118	84 (71.2%)	34 (28.8%)	

**Table 2:** Results of multiple regression analysis predicting a UTI caused by bacteria resistant to at least one antibiotic.

Variable	B	Std. Err. of B	β	*p* Value
Gender	0.103	0.089	0.103	0.253
Age	0.077	0.092	0.007	0.403
First episode of UTI	0.020	0.136	0.019	0.890
CAKUT	0.052	0.098	0.051	0.592
Neurogenic bladder	0.249	0.100	0.370	0.014
Previous antibiotic therapy for a UTI	0.031	0.132	0.031	0.811
Previous antibiotic therapy for other infection	0.041	0.084	0.091	0.629
Antibiotic prophylaxis for a UTI	−0.193	0.091	−0.258	0.036
Urological surgery in the past	0.217	0.092	0.256	0.021

B—unstandardized coefficient; Std. Err.—standard error; β—standardized coefficient.

**Table 3:** Number of records screened and included in the final analysis according to year of hospitalization.

Year of Hospitalization	Number of Records Identified During Initial Data Search	Number of Records Included in the Final Analysis
2018	65	40
2019	39	21
2020	45	26
2021	45	25
2022	48	28
Total	242	140

## Data Availability

The data presented in this study are available upon request from the corresponding author.

## References

[1] ‘t Hoen L.A., Bogaert G., Radmayr C., Dogan H.S., Nijman R.J.M., Quaedackers J., Rawashdeh Y.F., Silay M.S., Tekgul S., Bhatt N.R. (2021). Update of the EAU/ESPU guidelines on urinary tract infections in children. J. Pediatr. Urol..

[2] Leung A.K.C., Wong A.H.C., Leung A.A.M., Hon K.L. (2019). Urinary Tract Infection in Children. Recent Pat. Inflamm. Allergy Drug Discov..

[3] Simões E Silva A.C., Oliveira E.A., Mak R.H. (2020). Urinary tract infection in pediatrics: An overview. J. Pediatr. Rio J..

[4] Khan A., Jhaveri R., Seed P.C., Arshad M. (2019). Update on Associated Risk Factors, Diagnosis, and Management of Recurrent Urinary Tract Infections in Children. J. Pediatr. Infect. Dis. Soc..

[5] Boev C., Kiss E. (2017). Hospital-Acquired Infections: Current Trends and Prevention. Crit. Care Nurs. Clin. N. Am..

[6] Iacovelli V., Gaziev G., Topazio L., Bove P., Vespasiani G., Finazzi Agrò E. (2014). Nosocomial urinary tract infections: A review. Urologia.

[7] Raoofi S., Pashazadeh Kan F., Rafiei S., Hosseinipalangi Z., Noorani Mejareh Z., Khani S., Abdollahi B., Seyghalani Talab F., Sanaei M., Zarabi F. (2023). Global prevalence of nosocomial infection: A systematic review and meta-analysis. PLoS ONE.

[8] Medina-Polo J., Naber K.G., Bjerklund Johansen T.E. (2021). Healthcare-associated urinary tract infections in urology. GMS. Infect. Dis..

[9] Devrim F., Serdaroğlu E., Çağlar İ., Oruç Y., Demiray N., Bayram N., Ağın H., Çalkavur S., Sorguç Y., Dinçel N. (2018). The Emerging Resistance in Nosocomial Urinary Tract Infections: From the Pediatrics Perspective. Mediterr. J. Hematol. Infect. Dis..

[10] Mongkonsritragoon W., Anugulruengkitt S., Chanakul A. (2023). Incidence of healthcare-associated urinary tract infections in Thai children. Pediatr. Int..

[11] van Duin D., Paterson D.L. (2020). Multidrug-Resistant Bacteria in the Community: An Update. Infect. Dis. Clin. N. Am..

[12] Esposito S., Biasucci G., Pasini A., Predieri B., Vergine G., Crisafi A., Malaventura C., Casadio L., Sella M., Pierantoni L. (2022). Antibiotic Resistance in Paediatric Febrile Urinary Tract Infections. J. Glob. Antimicrob. Resist..

[13] Nabal Díaz S.G., Algara Robles O., García-Lechuz Moya J.M. (2022). New definitions of susceptibility categories EUCAST 2019: Clinic application. Rev. Esp. Quimioter..

[14] Alsaywid B.S., Alyami F.A., Alqarni N., Neel K.F., Almaddah T.O., Abdulhaq N.M., Alajmani L.B., Hindi M.O., Alshayie M.A., Alsufyani H. (2023). Urinary tract infection in children: A narrative review of clinical practice guidelines. Urol. Ann..

[15] Parry C.M., Taylor A., Williams R., Lally H., Corbett H.J. (2023). Antimicrobial resistance of breakthrough urinary tract infections in young children receiving continual antibiotic prophylaxis. Eur. J. Pediatr..

[16] Zhang K., Zhang Y., Chao M., Hao Z. (2023). Prevalence, Pathogenic Bacterial Profile and Antimicrobial Susceptibility Pattern of Urinary Tract Infection among Children with Congenital Anomalies of the Kidney and Urinary Tract. Infect. Drug Resist..

[17] Isac R., Basaca D.G., Olariu I.C., Stroescu R.F., Ardelean A.M., Steflea R.M., Gafencu M., Chirita-Emandi A., Bagiu I.C., Horhat F.G. (2021). Antibiotic Resistance Patterns of Uropathogens Causing Urinary Tract Infections in Children with Congenital Anomalies of Kidney and Urinary Tract. Children.

[18] Daniel M., Szymanik-Grzelak H., Sierdziński J., Podsiadły E., Kowalewska-Młot M., Pańczyk-Tomaszewska M. (2023). Epidemiology and Risk Factors of UTIs in Children-A Single-Center Observation. J. Pers. Med..

[19] Wasilewska A. Recommendations of Polish Society for Pediatric Nephrology in the Management of Children with the Urinary Tract Infection. Poland 2021. https://ptnfd.org/site/resource/1323,zalecenia-zum_2021.pdf.

[20] Vazouras K., Velali K., Tassiou I., Anastasiou-Katsiardani A., Athanasopoulou K., Barbouni A., Jackson C., Folgori L., Zaoutis T., Basmaci R. (2020). Antibiotic treatment and antimicrobial resistance in children with urinary tract infections. J. Glob. Antimicrob. Resist..

[21] Montagnani C., Tersigni C., D’Arienzo S., Miftode A., Venturini E., Bortone B., Bianchi L., Chiappini E., Forni S., Gemmi F. (2021). Resistance Patterns from Urine Cultures in Children Aged 0 to 6 Years: Implications for Empirical Antibiotic Choice. Infect. Drug Resist..

[22] Sorlózano-Puerto A., Gómez-Luque J.M., Luna-Del-Castillo J.D., Navarro-Marí J.M., Gutiérrez-Fernández J. (2017). Etiological and Resistance Profile of Bacteria Involved in Urinary Tract Infections in Young Children. BioMed Res. Int..

[23] Hrbacek J., Cermak P., Zachoval R. (2020). Current Antibiotic Resistance Trends of Uropathogens in Central Europe: Survey from a Tertiary Hospital Urology Department 2011–2019. Antibiotics.

[24] Dejonckheere Y., Desmet S., Knops N. (2022). A study of the 20-year evolution of antimicrobial resistance patterns of pediatric urinary tract infections in a single center. Eur. J. Pediatr..

[25] Werbel K., Jankowska D., Wasilewska A., Taranta-Janusz K. (2021). Clinical and Epidemiological Analysis of Children’s Urinary Tract Infections in Accordance with Antibiotic Resistance Patterns of Pathogens. J. Clin. Med..

[26] Michno M., Sydor A., Wałaszek M., Sułowicz W. (2018). Microbiology and Drug Resistance of Pathogens in Patients Hospitalized at the Nephrology Department in the South of Poland. Pol. J. Microbiol..

[27] Raupach T., Held J., Prokosch H.U., Rascher W., Zierk J. (2020). Resistance to antibacterial therapy in pediatric febrile urinary tract infections-a single-center analysis. J. Pediatr. Urol..

[28] Budnik T.V., Bevzenko T.B. (2020). A ten-year analysis of changes in the sensitivity of the leading uropathogen to antibacterial agents in children with urinary tract infection in the nephrology department. Wiad. Lek..

[29] Wanke-Rytt M., Sobierajski T., Lachowicz D., Seliga-Gąsior D., Podsiadły E. (2023). Analysis of Etiology of Community-Acquired and Nosocomial Urinary Tract Infections and Antibiotic Resistance of Isolated Strains: Results of a 3-Year Surveillance (2020–2022) at the Pediatric Teaching Hospital in Warsaw. Microorganisms.

[30] Esposito S., Maglietta G., Di Costanzo M., Ceccoli M., Vergine G., La Scola C., Malaventura C., Falcioni A., Iacono A., Crisafi A. (2021). Retrospective 8-Year Study on the Antibiotic Resistance of Uropathogens in Children Hospitalised for Urinary Tract Infection in the Emilia-Romagna Region, Italy. Antibiotics.

[31] Choi U., Kim E., Lyu D.H., Kim K.S., Park B.H., Chung H., Han C.H., Bae S. (2022). The change of antibiotic susceptibility in febrile urinary tract infection in childhood and adolescence during the last decade. Investig. Clin. Urol..

[32] Erol B., Culpan M., Caskurlu H., Sari U., Cag Y., Vahaboglu H., Özumut S.H., Karaman M.I., Caskurlu T. (2018). Changes in antimicrobial resistance and demographics of UTIs in pediatric patients in a single institution over a 6-year period. J. Pediatr. Urol..

[33] Autore G., Neglia C., Di Costanzo M., Ceccoli M., Vergine G., La Scola C., Malaventura C., Falcioni A., Iacono A., Crisafi A. (2022). Clinical Outcome of Discordant Empirical Therapy and Risk Factors Associated to Treatment Failure in Children Hospitalized for Urinary Tract Infections. Children.

[34] Jerardi K.E., Auger K.A., Shah S.S., Hall M., Hain P.D., Myers A.L., Williams D.J., Tieder J.S. (2012). Discordant antibiotic therapy and length of stay in children hospitalized for urinary tract infection. J. Hosp. Med..

[35] Wang M.E., Lee V., Greenhow T.L., Beck J., Bendel-Stenzel M., Hames N., McDaniel C.E., King E.E., Sherry W., Parmar D. (2020). Clinical Response to Discordant Therapy in Third-Generation Cephalosporin-Resistant UTIs. Pediatrics.

[36] Sihra N., Goodman A., Zakri R., Sahai A., Malde S. (2018). Nonantibiotic prevention and management of recurrent urinary tract infection. Nat. Rev. Urol..

[37] Meena J., Thomas C.C., Kumar J., Raut S., Hari P. (2021). Non-antibiotic interventions for prevention of urinary tract infections in children: A systematic review and meta-analysis of randomized controlled trials. Eur. J. Pediatr..

[38] Sadeghi-Bojd S., Naghshizadian R., Mazaheri M., Ghane Sharbaf F., Assadi F. (2020). Efficacy of Probiotic Prophylaxis after the First Febrile Urinary Tract Infection in Children with Normal Urinary Tracts. J. Pediatr. Infect. Dis. Soc..

[39] Hosseini M., Yousefifard M., Ataei N., Oraii A., Mirzay Razaz J., Izadi A. (2017). The efficacy of probiotics in prevention of urinary tract infection in children: A systematic review and meta-analysis. J. Pediatr. Urol..

